# The role of inflammation in age-related disease

**DOI:** 10.18632/aging.100531

**Published:** 2013-01-30

**Authors:** T. Kevin Howcroft, Judith Campisi, Germaine Buck Louis, Martyn T. Smith, Bradley Wise, Tony Wyss-Coray, Alison Deckhut Augustine, Janet E. McElhaney, Ron Kohanski, Felipe Sierra

**Affiliations:** ^1^ Division of Cancer Biology, NCI, NIH, Bethesda, MD 20892, USA; ^2^ Buck Institute for Research on Aging, Novato, CA 94945, USA and Lawrence Berkeley National Laboratory, Berkeley, CA 94702 USA; ^3^ Office of the Director, NICHD, NIH, Bethesda, MD 20892, USA; ^4^ Division of Environmental Health Sciences, University of California, Berkeley, CA 94720, USA; ^5^ Division of Neuroscience, NIA, NIH, Bethesda, MD 20892, USA; ^6^ Department of Neurology and Neurological Sciences, Stanford University School of Medicine, Stanford, CA 94305; ^7^ Division of Allergy, Immunology and Transplantation, NIAID, NIH, Bethesda, MD 20892, USA; ^8^ Advanced Medical Research Institute of Canada, Sudbury, ON, P3E 5J1, Canada; ^9^ Division of Aging Biology, NIA, NIH, Bethesda, MD 20892, USA

## Abstract

The National Institutes of Health (NIH) Geroscience Interest Group (GSIG) sponsored workshop, The Role of Inflammation in *Age-Related Disease*, was held September 6th-7th, 2012 in Bethesda, MD. It is now recognized that a mild pro-inflammatory state is correlated with the major degenerative diseases of the elderly. The focus of the workshop was to better understand the origins and consequences of this low level chronic inflammation in order to design appropriate interventional studies aimed at improving healthspan. Four sessions explored the intrinsic, environmental exposures and immune pathways by which chronic inflammation are generated, sustained, and lead to age-associated diseases. At the conclusion of the workshop recommendations to accelerate progress toward understanding the mechanistic bases of chronic disease were identified.

## INTRODUCTION

The NIH Geroscience Interest Group (GSIG) sponsored workshop, *The Role of Inflammation in Age-Related Disease***,** was held September 6^th^-7^th^ in Bethesda, MD. The workshop opened with a plenary talk by **Luigi Ferrucci (NIA, NIH)** who presented an overview of the literature supporting the emergence a mild pro-inflammatory state that is closely linked to the major degenerative diseases of the elderly. The focus of the workshop was to better understand the origins and consequences of this low level chronic inflammation in order to design appropriate interventional studies aimed at improving healthspan. For many, inflammation is simply understood as a trajectory of biomarkers, for example the appearance of IL-6 or C-reactive protein (CRP), associated with a disease. However, inflammation is a very complex response to an injury, infection, or other stimulus, in which many different cells types and secreted factors orchestrate protective immunity, tissue repair, and resolution of tissue damage. Whereas acute inflammation limits tissue damage and resolves, chronic prolongation of the inflammatory state leads to progressive tissue damage. A central question, then, is how do we describe and begin to understand the mild pro-inflammatory state of aging. Among the causal pathways linked to the major diseases associated with aging, including physical frailty, are changes in body composition, energy imbalance, homeostatic dysregulation, and neurodegeneration. Chronic inflammation is strongly connected with each of these aging phenotypes. The inflammatory mediators IL-6, IL-18, and CRP increase with age in both women and men and are highly correlated with obesity and degenerative disease. Muscle strength, as measured by walking speed, also correlates with circulating IL-6 levels. Individuals with the lowest circulating levels had the highest walking speed. Further, in mouse models, reducing inflammation through ablation of the β subunit of the inhibitor of NFκB kinase (IKK), IKKb, improved skeletal muscle strength, maintained mass, and promoted regeneration. These data suggest that inflammation blocks critical metabolic signals that support muscle maintenance. In addition, anti-inflammatory drugs ameliorate type II diabetes and prevent cognitive decline in humans. In mouse models, blocking the NFκB pathway prevented insulin resistance and improved glucose metabolism. Lastly, systemic inflammation may predispose the microglia to a pro-inflammatory state that is associated with neurodegeneration. Although it is not clear what causes age-associated chronic inflammation, possible mechanisms include a disregulated NF-kB pathway, impaired mitochondrial function leading to excessive reactive oxygen species (ROS), the accumulation of senescent cells, and a decline in autophagy with age. Whether reducing inflammation will lead to beneficial effects on human health and function is the defining biological and medical challenge of the next decade. Following the plenary presentation were four scientific sessions, each considering in greater depth the relationships between aging, chronic inflammation, and the development of chronic diseases.

### Session I: Cellular Senescence, Inflammation, and Cancer

The first session focused on the interconnections between cellular senescence, inflammation, and cancer. **Judith Campisi (Buck Institute for Research on Aging and Lawrence Berkeley National Laboratory), session Chair,** presented data supporting the hypothesis that cellular senescence and its resulting secretion of inflammatory mediators are common factors underlying the two classes of age-associated diseases, degenerative decline and cancer (the latter being a hyperplastic response). Senescent cells accumulate with age, are found in increased numbers at sites of pathologies associated with aging, and secrete an array of inflammatory factors, chemokines, matrix metalloproteinases, and growth factors designated the senescence-associated secretory phenotype (SASP). Senescence is a cellular response triggered by stress that likely co-evolved as a mechanism to prevent cancer, as well as to repair damaged tissue. For example, irradiating mice causes an accumulation of senescent cells, which promote increased growth and metastasis of transferred tumor cells. In contrast, conditional deletion of senescent cells reduced both primary and tumor metastases. These data suggest that the SASP can directly degrade the cancer niche and promote cancer progression. Senescent cells are also produced very rapidly at the sites of wounding and their conditional deletion significantly delays wound healing. This dual purpose of the SASP is also evident in the ability of senescent cells to induce an epithelial-mesenchymal transition (EMT), which allows epithelial cells to participate in wound healing, but is also an important part of cancer progression. However, precisely which components of the SASP are drivers of inflammation and chronic disease is not clear. Although circulating IL-6 and CRP are the most frequently measured biomarkers of inflammation, there is an urgent need for a more comprehensive profile of inflammation across individuals and across lifespan. Another critical issue is to determine whether the degraded niches seen in degenerative pathology are similar to the degraded niches in hyperplastic disease.

Beginning with the observation that caloric restriction and obesity have opposite effects on inflammation and lifespan, **Jim Kirkland (Mayo Clinic)** addressed the role of adipose tissue in inflammation- and age-associated chronic disease. Adipose, for many people, is the single largest organ and contains the largest pool of multipotent progenitor cells in the body. As adipose tissue dysfunction increases with age, there is an associated impairment in adipogenesis and accumulation of senescent preadipocytes. Senescent preadipocytes and the immune cells they attract secrete pro-inflammatory cytokines, including TNFa and IL-6, which potently repress the transcription factors PPARγ and C/EBPa, which are mainly responsible for adipogenesis and are known to decrease with age. Long-lived mouse strains accumulate less senescent cells, either due to decreased generation or increased clearance, and have adipose tissue that resembles younger animals. These data led to studies to examine the effect of conditional deletion of senescent preadipocytes on disease progression. The loss of senescent cells could delay but not reverse age-related adipose tissue and muscle dysfunction in a progeroid mouse model, as do drugs that interfere with the secretory phenotype of senescent cells. Thus, there is a critical need to identify novel biomarkers (both blood and imaging) of the burden of senescent cells *in vivo* and drugs that target their pro-inflammatory secretory phenotype or remove senescent cells in murine age-related disease models and clinical trial paradigms.

**Michael Karin (UCSD)** further explored the connections between obesity, inflammation, and defective autophagy associated with the development of hepatocellular carcinoma (HCC) and pancreatic cancer. The effect of obesity on chemically-induced HCC was modeled by treating mice with carcinogens and then placing them on high-fat or low-fat diets. Mice on a high-fat diet had increased tumor burden that was triggered by enhanced expression of TNFa and IL-6 inflammatory cytokines, which sustained hepatosteatosis and myeloid infiltration and lead to Stat3 activation in HCC progenitors. Accordingly, liver-specific Stat3 knockout mice are relatively resistant to HCC induction. Mice on the high-fat diet also accumulated the protein chaperone p62 in vacuoles suggesting that obesity impaired autophagy, a cellular process that clears p62 bound to misfolded proteins trough lysosomal degradation. In addition to HCC, the incidence of pancreatic cancer is also increasing in the United States and on autopsy many people have precancerous pancreatic lesions with a mutated Kras oncogene. Risk factors for pancreatic cancer include chronic pancreatitis, obesity, type II diabetes, smoking, and old age. Pancreatic-specific inhibition of the α subunit of IKK, to generate IKKα null mice is a useful model for chronic pancreatitis and pancreatic cancer. Loss IKKα leads to pancreatitis, creates a fibrotic and inflammatory tumor microenvironment, and accelerates the progression of precancerous lesions to pancreatic cancer. The latter is seen in mice that are both IKKα null in pancreatic epithelial cells and express an activated (mutant) Kras^G12D^. In addition, p62 accumulates in acinar cells from IKKα knockout mice due to defective lysosomal degradation. Accumulation of p62 leads to formations of ubiquitinated protein aggregates suggesting a defect in autophagy, which cause endoplasmic reticulum (ER) stress and accumulation of ROS. Genetic deletion of p62 protects pancreas-specific IKKα null mice from development of chronic pancreatitis although it does not correct the autophagy defect. In addition, p62 ablation slows down the progression of pancreatic cancer. Taken together, these data suggest a model whereby obesity impairs autophagy leading to ER stress in acinar cells, resulting in oxidative damage leading to metaplasia.

**Pete Nelson (FHCRC)** discussed how DNA damaging therapeutics led to profound increases in many inflammatory pathways in the prostate. The increase in inflammatory cytokines could be attributed, at least in part, to infiltrating immune cells. However, the normal stromal cell component of the tumor microenvironment, which accumulates therapy-induced damaged and senescent cells, also contributed. Thus, the fundamental nature of the inflammatory environment generated by cytotoxic cancer treatment is similar to that of the SASP phenotype, only the magnitude is larger than that observed during normal aging. Interestingly, the incidence of prostate cancer increases with age, as does inflammation, while the effects of androgens on the prostate wane with age. Thus, it is important to consider the effects of hormones in general, and androgens in particular, on prostate cancer progression and inflammation in the context of aging. In the human prostate, androgen withdrawal leads to a marked influx in inflammatory immune cells. Precisely why inflammatory cells increase in the prostate is not clear but this finding suggests that there is a connection between androgen levels, inflammation, and cancer. One clue as to mechanism is that androgens are known to affect the permeability of the blood-testis barrier through regulating the integrity of tight junctions. Claudins are the principal molecules that regulate cell-cell adhesion at tight junctions and are expressed at very low levels in men with low testosterone levels. Supplementing testosterone restored claudin expression and the loss or gain of claudins was correlated with relative presence or absence of inflammatory infiltrates in the prostate, respectively. These findings suggest a model whereby testosterone regulates an age-associated tumor suppressive program operating through the prostate tissue microenvironment. Key questions that remain are to determine whether systemic or local inflammation effects are dominant and whether the elevated inflammatory status in the prostate is due to impaired tight junctions and the loss of immune privilege secondary to androgen deficiency.

In summary, senescent cells or the SASP that accumulate with increasing age, or as a result of radio/chemotherapy, could provide a common framework for the development of degenerative diseases and cancer. However, whether disease status is dictated by the target cells or their niches or both is not clear.

### Session I: Research Opportunities

- Determine the prime cause (or causes) of age-related chronic inflammation.
- Assess whether chronic inflammation is a driver of age-related disease or a responder to one or more prime causes of aging.
- Determine pathways by which chronic inflammation damages macromolecules or disrupts tissue homeostasis in the context of age-related disease.
- Assess the relative contributions of local versus systemic inflammation in driving aging phenotypes and age-related pathology.
- Identify interventions (small molecules, antibodies, or life style) that can modulate or eliminate the sources of chronic inflammation.
- Determine the senescence/damage responses of different mammalian cell types, including mitotic (including stem and progenitor cells) and post-mitotic cells.
- Develop an atlas of when and where senescent cells arise across the life-span and during the development and progression of various age-related diseases.
- Catalog how circulating inflammatory factors and immune infiltration of specific tissues vary across the life-span and during the development and progression of age-related diseases.

### Session II: Lifespan Exposures and Inflammation-Associated Diseases of Aging – Exposome Paradigm

The second session talks explored the exposome paradigm and its applicability for assessing the impact of environmental exposures on inflammation-associated diseases across the lifespan. The exposome is defined as the totality of environmental exposures from (pre)conception onwards, and is conceptualized to complement study of the genomic influences on health and disease. **Martyn Smith (University of California)** led a discussion on the multitude of challenges of measuring the environmental component of gene-environment interactions. Although advances in DNA sequencing now permit rapid, accurate, and high throughput identification of the genetic component of this interaction, the quantitative assessment of the environmental component is much more challenging reflecting their dynamic and hierarchical nature including during sensitive or vulnerable periods of life. While many environmental exposures have valid and reliable biomarkers (e.g., ionizing radiation, persistent organochlorine pollutants), others have no available ‘biomarkers’ requiring proxy exposure assessments (e.g., poverty, physical activity), which may be subject to measurement error. Still, marked technologic advances offer various monitors and sensors that might improve measurement of exposures. The attributable risks for environmental exposures such as lifestyle, occupation and diet and a spectrum of diseases are notable, and many are amenable to public health intervention. Environmental exposures may damage nucleic acids, proteins, and lipids inside the body underscoring the importance of characterizing the exposome. Thus, for human health ‘environmental exposure’ relates to levels of toxic chemicals (from all sources) in the internal environment. This could be called the ‘internal exposome’ and it is an appropriate place to begin untargeted research that might lead to clues about external exposures. This internal exposome would reflect not only chemicals coming from the external environment but those generated endogenously through processes such as inflammation. Chronic inflammation is a source of reactive oxidants and electrophiles in addition to cytokines and other biological mediators. Thus, the exposome provides a conceptual framework to enable the measurement of the entirety of chemicals, both exogenous and endogenous, throughout the lifespan of an individual. Further, the exposome research paradigm expands our view of the environment to include all non-genetic factors experienced by individuals throughout life. Some recent ‘proof of concept’ studies utilizing metabolomics have found differences in the metabolomes of children with autism compared to their unaffected siblings. This idea needs more exploration including expanding the measurement of the chemical universe.

**Peter Dedon (MIT)** discussed emerging concepts concerning the chemical nature of inflammation and the chemical mechanisms linking inflammation to cancer. Clearly, there are different types of inflammation including immune cell-dependent and immune cell-independent pathways that involve the generation of cytokines and reactive oxygen species. In addition, both locally- and systemically-generated inflammatory factors can cause damage either at the site of production or at some distance. This leads to a central question of what endogenous exposures drive chronic inflammatory processes and do the different types of inflammation generate a continuum of common pathologies. Chronic infection with the pathogen *Helicobacter pylori* can lead to gastric cancer. The precise mechanistic links by which chronic exposure to infectious agents leads to chronic inflammation and cancer are not fully understood. Tissue damage provokes the influx of immune cells that generate inflammatory cytokines and reactive molecules such as reactive oxygen species (ROS) including superoxide and reactive nitrogen species (RNS) including nitric oxide (NO) that damage of all kinds of macromolecules including nucleic acids, proteins, lipids, and various metabolites. The complexity of endogenous highly reactive chemicals during an infection is an example of the need to be able to assess internal chemical exposures including cytokines and chemokines. Many reactive species, such as NO, at low but physiologically relevant concentrations act as potent signaling molecules. In contrast, high NO concentrations such as those made by activated macrophages, cause extensive damage to nearby biomolecules leading to mutation, cell death and tissue damage. Thus, the endogenous internal concentrations of these inflammatory chemicals are critical for understanding their roles in health and disease. However, we do not have good knowledge of the effects of most inflammatory mediators in this gray zone between low and high concentrations. Furthermore, some of the breakdown products of these pathways are short-lived. Thus, determining the effect of age-related NO production is a highly complicated question. In some instances, the types of damage can be used as a specific biomarker of the chemical environment. For example, protein and DNA adducts and other damage products can be measured simultaneously to provide a survey of the chemical environment related to pathology. This approach can be applied to examine the links between inflammation (colitis) and colon cancer in RAG2 knockout model. RAG2 deficient mice do not develop mature T and B lymphocytes and when infected with *Helicobacter hepaticus* develop all the progressive stages of colorectal carcinoma (CRC). Infection with *H. hepaticus* leads to destructive colitis associated with the influx of neutrophils and macrophage. Array analyses indicated that 173 response genes discriminated between controls and early and late disease. Both ROS and RNS generating genes were massively upregulated in the colon. However, if production of NO is inhibited, these mice will not develop CRC nor inflammation. This study establishes the connection between immune inflammation, NO, and cancer. However, a survey of the damage products has led to other interesting questions. Whereas all major DNA repair pathways were shut-down in the colon, these were increased in the liver and there is more evidence of damage in the liver than in the colon. Lastly, halogenation damage products are emerging as better predictors of chronic inflammatory damage than previously studied DNA or RNA adducts.

**Larry Marnett (Vanderbilt)** focused on the application of mass spectrometry for profiling electrophile conjugates that are generated during inflammation. Various xenobiotic and endobiotic metabolites are converted to reactive electrophiles capable of modifying cellular macromolecules perturbing their normal activities. Relating electrophile exposure to disease etiology requires a comprehensive profile of the types and amounts of electrophiles adducted to cellular macromolecules. For example, serum albumin can be used as an electrophile trap to quantitate the number of electrophiles capable of generating unique modifications to albumin. In order to profile the effects of electrophiles generated during inflammation, technology must be developed that can qualitatively and quantitatively analyze classes of electrophile adducts in a global fashion. What electrophiles are generated during inflammation, how they react with their substrates, and whether the formation of conjugates can be measured in cellular extracts, intact cells or tissues are major questions that need to be addressed. Mass spectrometry is well-suited as an analytical platform to provide an exposome perspective of the electrophiles generated during an inflammatory response and oxidative stress. Reference proteomes in THP1 macrophage and RKO cancer cell lines were compared to protein targets of the lipid oxidation products, alkynyl-4-hydroxynonenal (aHNE) and alkynyl-4-oxononenal (aONE). Proteome signatures that reflect modification by aHNE, aONE, both, or neither could be generated. Further, while some proteins were hypersensitive to modification by either aHNE or aONE even at low concentrations others were only modified at high concentrations of electrophile. Thus, there are differences in the sensitivity of damage that may be due to how the cells handle specific electrophiles. Future studies can begin to ask what types of damage are induced in intact tissues during normal physiological processes or disease using Mass Spectrometry-based imaging approaches. Mass spectrometry- based imaging complements targeted *in vitro* approaches for detecting electrophile conjugates in normal and diseased tissues in an unbiased fashion. Thus, focusing on the internal environment may provide a snapshot of cumulative of both external and internal exposures that represent an individual's exposome.

**Bob Hiatt (UCSF)** reflected on the potential of the exposome concept for furthering epidemiological research. The current approach, described as a bottom-up approach, typically measures individual or a small set of exposures in relation to a disease endpoint. Although this approach continues to be important, it has inherent limitations. Given that there are at minimum 87,000 well-characterized industrial chemicals worldwide, it is challenging to identify and estimate all health effects associated with any one element let alone the effect(s) of complex chemical mixtures across the lifespan. Further, some chemicals have short half-lives that make linking disease phenotypes to specific exposures difficult, yet these transient chemicals can have significant and lasting biological effects. Relevant exposures go beyond toxicants (such as chemical contaminants and environmental pollutants) and include radiation, infectious agents, diet, tobacco, alcohol, and medical interventions. Further, additional exposures such as social capital, education level, financial resources, stress, noise, heat, and indigenous climate can affect human health. The concept of the exposome that evaluates the internal environment and uses a “top-down” approach offers an exciting shift to the current paradigm that seeks to measure the cumulative environmental exposure. If such an approach can be adapted to large-scale human studies, it will potentially enable unbiased approaches to assess the effects of environment in health and disease at the population level. For epidemiologists, it is critical to understand the meaning of abnormalities in the internal environment that influence population health and are amenable to public health intervention. If suitable disease biomarkers can be defined and linked to specific exposures, such studies would inform the design and implementation of both etiologic and intervention research. Thus, there is a critical need for pioneering efforts to apply the concept of the exposome to large-scale epidemiologic studies to establish its feasibility and promise. However, there are many issues that need to be clarified before embarking on such studies, including the identification and availability of biomarkers for a multitude of exposures and health outcomes, whose validity and reliability have been empirically demonstrated. Also, it will be important to measure biomarkers during different developmental and stressful life stages and events such as puberty, pregnancy, major illness, or new social situations that may affect exposures and the effects of those. Advances in this area of exposure science need to remain flexible enough to encompass new knowledge and technologies.

### Session II: Research Opportunities

- Determine the relevant environmental exposures associated with disease status.
- Expand the exposome beyond serum and blood to include CSF, amniotic fluid, saliva, urine, feces, tears, and tissue samples.
- Expand the serum protein adductome to encompass the totality of modifications of serum proteins and identify adducts on tissue proteins.
- Expand the metabolome to include the diet (food metabolome) and xenometabolome, and improve the annotation.
- Expand the receptor-ome to include cell-based assays for other receptors (e.g. PPAR, CAR etc.) and the chemicals they interact with.
- Increase the number of metals that can be measured simultaneously and differentiated (metallomics).
- Identify novel biomarkers of past exposures to infectious agents and environmental or psychological stress (including epigenetics).
- Encourage the development of interdisciplinary research teams including epidemiology, medicine, toxicology, exposure science, analytical chemistry, bioengineering and advanced bioinformatics and biostatistics.
- Accelerate the development of exposome tools including mass spectrometers with enhanced analytical chemistry capabilities and microfluidics.
- Encourage exposome pilot studies:
  - Focus on extremes, obese vs. lean old v. young; smokers v. non-smokers,
  - Study migrant population,
  - Intervention studies allowing for repeated sampling,
  - Repeat sampling of pregnant women

### Session III: Inflammation and Neurological Diseases of Aging

**Tony Wyss-Coray (Stanford University)**, session Chair, started off the session by addressing the role of systemic inflammation in brain aging and neurodegeneration. Age-related decline in cognitive function is an issue for the elderly and age is a key risk a factor for Alzheimer's disease (AD). Brain aging in mice is associated with decreases in learning and memory, synaptic plasticity, and adult neurogenesis (i.e. the formation of new neurons), while indices of neuroinflammation increase. Inflammatory processes in the brain are mainly mediated by the intrinsic innate immune system consisting of astrocytes and microglial cells and cytokine, chemokine and growth factor signaling molecules. Though markers of microglia activation increase with age, it remains unclear what is the function of activated microglia, whether there are subtypes of microglia with different functions, and whether activation is causative or reactive to neurodegeneration. Recent genome-wide association studies implicate innate and adaptive immune response genes as risk factors for neuroinflammation and subsequent pathogenesis associated with AD. **Wyss-Coray** posed the provocative questions: Are neurodegenerative diseases systemic diseases and are systemic changes a consequence of brain degeneration or vice-versa? Parabiosis, in which the circulatory systems of two mice are joined, provides an experimental paradigm to address these questions: joining the blood supply of young and old mice allows for the examination of the effects of an old systemic environment on brain function in the young and vice versa. In such heterochronic parabionts, neurogenesis, synaptic activity and memory function were decreased in young mice when exposed to an old systemic environment. This ‘aging’ effect was reproduced by injection of plasma from old animals into young mice, and proteomic analysis of blood revealed several factors that increased in young heterochronic parabionts as well as in normal aging mice. One such factor, the chemokine CCL11 (or eotaxin), reduced adult neurogenesis and learning and memory in young mice when administered systemically. Quite interestingly, exposure of an old brain to a young systemic environment produced a more youthful phenotype: increased neurogenesis, synaptic activity, and memory with reduced neuroinflammation. The search is now on to identify systemic ‘rejuvenating’ factors, and additional aging factors. The finding that systemic plasma factors can modulate neurogenesis, microglial activation and cognition opens the door to defining a set of secretory signaling proteins that may be useful in the discovery of biomarkers and therapeutic agents in brain aging and disease.

**Richard Ransohoff** (**Cleveland Clinic Foundation)** explored the function of brain macrophages, derived from microglia or monocytes, in neurodegeneration using experimental autoimmune encephalomyelitis (EAE) as a model of CNS inflammation. Microglia are self-renewing myeloid cells found in the CNS parenchyma from early embryonic life, while monocytes originate in the bone marrow and circulate in the bloodstream. Macrophages of the CNS are activated myeloid cells which originate from either monocytes or microglia. Monocytes, microglia, and their activated macrophages can be distinguished by the chemokine receptors that they express: microglia express the chemokine receptor CX3CR1, while monocytes are positive for CCR2 which is required for their entry into the brain. Though monocytes and microglia live parallel but separate lives during adulthood, they can meet in the inflamed CNS as macrophages. Macrophage capacities for harmful neuroinflammation or beneficial tissue repair could depend on their origin from microglia or infiltrating monocytes, as well as on the underlying pathological process. Labeling of monocytes and microglia with different fluorescent proteins in transgenic mice allowed monitoring of their activation and function in the EAE model of CNS inflammation and demyelination. Immuno-electon microscopy (EM) of labeled cells also identified a morphological difference between microglia and monocytes such that microglia exhibited round nuclei, while monocytes showed folded and irregular shaped nuclei. This morphological characteristic was used in serial block-faced scanning EM of EAE tissue, in which a single axon and surrounding cells could be analyzed, to show that monocytes invade myelin nodes and mediate demyelination, while microglia clear myelin debris. So, in this model, monocytes are detrimental due to myelin stripping and microglia are beneficial via phagocytosis of debris. Monocytes and microglia exhibit differential patterns of gene expression in healthy animals as well as during the peak phase of EAE symptomology. Thus, monocyte-derived and microglia-derived macrophages can be distinguished and exert different functions in the CNS.

The theme of microglia as mediators of inflammatory processes continued with the presentation by **Thomas Montine(University of Washington)** on apolipoprotein E (ApoE) allele-specific regulation of innate immunity in brain. The ApoE protein is a pleiotropic molecule with actions in lipid transport, beta-amyloid (Aβ) trafficking, synaptic function, immune regulation, and intracellular signaling. Humans have three common alleles of the APOE gene called ε2, ε3, and ε4 alleles that encode protein isoforms differing in amino acids at two positions in the ApoE protein. The ε4 allele is the ancestral and AD high-risk form while also being associated with cognitive impairment, traumatic brain injury (TBI), stroke outcome, AIDS progression and susceptibility to infections. The ε3 allele is the most common and the neutral allele, and the ε2 allele is associated with decreased risk of AD. To begin to define the molecular mechanisms and function of the different APOE alleles in brain immunity, mice with targeted replacement of their APOE gene with human APOE alleles were generated. Activation of microglia, but not astrocytes, from mice expressing the human APOE4 gene resulted in increased secretion of TNF-α and IL-1β compared to APOE2 and APOE3 microglia. Microglia from APOE4 mice exhibited lower migration than APOE3 microglia in response to the C5a and ATP migratory stimuli, as well as decreased phagocytosis of FITC-Aβ. In the hippocampus (ex vivo and in vivo) of APOE4 mice, there were signs of synaptodendritic injury manifested as decreases in the post-synaptic marker PSD95 and retraction of dendritic arbors. These synaptic changes were blocked by an NSAID and by an inhibitor of p38 MAPK. A proinflammatory state (increased TNF-α, decreased IL-10 and microglia activation) was seen when monocytes from APOE4 mice were grafted into the brains of transgenic AD mouse models. As mentioned above, the APOE alleles are pleiotropic with varying contributions to the risk of age-related diseases, particularly AD. These studies suggest that one action of APOE is regulation of brain innate immunity, where the APOE4 allele may cause a proinflammatory microglial state and impaired microglial cell clearance of toxic molecules. Both of these effects may contribute to immune-mediated synaptodendritic damage, which is seen in brain aging and AD.

The last speaker of this session, **Malú Tansey (Emory University)**, highlighted research on the role of ceramide and sphingolipid signaling in TNF-dependent neurodegeneration in models of Parkinson's disease (PD). She began with a summary of the multifaceted actions of the two bioactive tumor necrosis factor (TNF) ligands (membrane-bound and soluble forms) and their two receptor subtypes in proliferation, inflammation and cell death, and with an overview of the symptoms, pathology and risk factors (genetic and environmental) of PD. Neuroinflammation is believed to be a major contributory factor in sporadic PD, with the inflammatory cytokine TNF implicated in dopamine-dependent neuron death. TNF signaling induces robust neurodegeneration in neurotoxin and endotoxin models of PD. Selective inactivation of soluble TNF signaling by a dominant-negative TNF variant (DN-TNF) rescued dopamine neurons from neurotoxin-induced death *in vitro*, while neutralization of soluble TNF by viral delivery of DN-TNF attenuated dopamine neuron loss in an in vivo rat model of PD. Even delayed administration of DN-TNF gene therapy in the rat PD model halted the progressive loss of dopamine neurons. These findings suggest that selective targeting of soluble TNF in early stages of PD may delay onset of disabling motor symptoms. Downstream effectors of soluble TNF-dependent neurotoxicity may be targets for therapeutic intervention and identification of such effectors focused on the sphingolipid ceramide signaling pathway. Soluble TNF induced ceramide accumulation in dopamine neurons and ceramide was neurotoxic towards dopamine neurons. Since ceramide is generated from sphingomyelin by the enzyme sphingomyelinase, enzyme inhibitors should block TNF action if ceramide is the key intracellular signaling molecule. Indeed, sphingomyelinase inhibitors attenuated TNF-induced neuronal death, ER stress, mitochondrial dysfunction, caspase 3 apoptotic signaling, and down-regulation of the p-Akt pathway in dopamine neurons. Soluble TNF also stimulated the accumulation of atypical sphingoid bases which compromised dopamine neuron survival. Soluble TNF interaction with its receptor TNFR1 may be a target for TNF antagonists like the DN-TNF peptide that could be administered via a gene therapy approach. Alternatively, the TNF-dependent activation of sphingomyelinases triggers production of ceramide and other downstream sphingolipid metabolites that promote caspase-dependent degeneration and cell death of dopamine neurons. Such metabolites, like the sphingoid bases, may represent novel drug targets for development of neuroprotective strategies to delay or attenuate the progressive loss of dopamine neurons that occurs in patients with PD.

Speakers in this session recognized age as a key risk factor for major neurodegenerative diseases with activation of innate immune pathways in brain and periphery being closely associated with neurodegeneration.

### Session III: Research Opportunities

- Consider age and/or the milieu of the aged brain and the influence of the systemic immune response (and systemic inflammation) as a parameter in neurodegenerative disease studies.
- Determine whether senescent cells accumulate in the CNS with age or disease status, which cell types undergo senescence and the contribution of senescent cells to neuroinflammation and neurodegeneration.
- Characterize microglia with age to assess whether microglia are harmful or beneficial in times of brain stress, injury and inflammation in the context of aging.
- Determine whether peripheral monocyte lineage cells are recruited into the aging CNS, and if so, where they home and what activities they mediate.
- Determine the interplay between microglia and monocytes and whether their complementary interactions change in the aging brain milieu.
- Characterize changes in the blood brain barrier (BBB) with age. How the BBB is maintained (including the roles of pericytes, astrocytes, and endothelial cells) and whether a leaky BBB contributes to neuroinflammation.
- Characterize the exposome (including proteomic and metabolic changes) of CSF in normal aging and neurodegeneration.

### Session IV: Immunosenescence, Inflammation, and Age-Associated Disease

**Janet McElhaney(Advanced Medical Research Institute of Canada)**, session Chair, opened the session with an overview of the effects of acute respiratory illness triggered by influenza, cytomegalovirus (CMV/ HHV-5) infection status, and chronic disease on inflammation and frailty. Although influenza is the single most vaccine preventable disease and vaccination programs are cost effective, influenza and complications of influenza infection remain a serious issue for the elderly, resulting in hospitalization in many cases. This is a serious issue since one in three older adults admitted to an acute care hospital for any reason are discharged at a higher level of disability than they had prior to their hospital stay. The increased susceptibility of the elderly to influenza infection and illness can be attributed to alterations in immune function that decrease vaccine efficacy and increase the risk of influenza infection. For example, numerous studies have shown defects in both adaptive and innate immunity in older individuals. In addition, older individuals who are sero-positive for CMV compared to CMV-negative elderly, exhibit poor antibody responses to influenza vaccination and an accumulation of terminally differentiated CD8+ T cells (largely driven by the response to persistent CMV infection); these CD8+ T cells express granzyme B, which has toxic effects when non-specifically released into the extracellular space in response to an inflammatory stimulus. High baseline levels of granzyme B in CMV-positive individuals are associated with a poor granzyme B response to influenza challenge, and may negatively impact on protection against serious influenza illness and the response to influenza vaccination when compared to CMV-negative elderly. An increase in granzyme B expression in influenza-specific T cells in response to influenza challenge in older individuals has been correlated with improved immunity to influenza. Further studies are needed to show the direct effect of CMV serologic status on influenza outcomes in this population. In contrast, increased granzyme B production in CMV-positive individuals occurs in the resting state, may be derived from both immune (e.g., chronically stimulated CMV-specific T cells) and non-immune cells (e.g., chondrocytes, keratinocytes), and contributes to increased inflammation and tissue destruction in response to injury. For example, release of granzyme B into the extracellular space cleaves Decorin, a cellular/pericellular matrix proteoglycan component of connective tissue, appears to participate in collagen disorganization, fibrosis, and tissue damage, which may lead to such conditions as increased frailty, skin tears, and aneurysms.

**Rita Effros (UCLA)** noted that the changes seen in the immune system of elderly individuals are comparable to those observed in younger HIV-infected individuals. For example, telomere length in both memory and naïve CD8+ T cell populations from HIV-infected individuals is comparable to the telomere lengths of similar cell subsets from individuals who are decades older. Moreover, persons with shortened leukocyte telomeres at age 60 have an eight-fold greater risk of subsequently dying from infection as compared to those with longer telomeres at age 60; and telomerase appears to be turned off in chronically stimulated T cells. In addition, chronically activated T cells contribute to the pro-inflammatory milieu in both elderly and HIV-infected young populations, and an abundance of “senescent” T cells correlates with multiple deleterious outcomes in both populations. For example, senescent CD8+ T cells, which exhibit increased expression of p16 and p21, also secrete factors such as TNF-alpha, Interleukin-6, and RANKL which are known to stimulate bone-resorbing osteoclasts, and may thereby contribute to increased bone loss in HIV-infected and elderly individuals. Obesity also contributes to bone loss; splenic T cells from obese mice exhibit increased expression of TNF-alpha, Interleukin-6, and Interleukin-1 beta, and bone marrow from these mice have increased proportions of activated memory T cells. Adipose-resident T cells from elderly mice exhibit similar cytokine production patterns, which contribute to the pro-inflammatory milieu and the development/progression of bone loss, cancer, and other chronic conditions of aging.

**Janko Nikolich-Zugich(University of Arizona)** discussed the roles of persistent infection and gut flora on the maintenance of immune defenses with age. In humans, persistent infections (CMV most strongly; other Herpesviruses less strikingly) are associated with manifestations of immune aging, reduced lifespan, and increased incidence of cardiovascular diseases. The conventional view of T cell aging suggests that there is a reduction in the number of naïve T cells and an increase in the memory T cell compartment. However, few studies have defined the precise changes in absolute T cell numbers in elderly individuals, or evaluated the effects of chronic CMV infection on “immunological” age. Dr. Nikolich-Zugich conducted a cross-sectional immune analysis of 150 CMV-negative and 244 CMV-positive adults (age 21-100), in which he defined naïve, effector, and memory profiles of CD4 and CD8 T cells; T cell receptor diversity; and CMV titer. These studies suggested that adult humans lose CD8^+^ T cells, but not CD4^+^ T cells, as they age. This loss was independent of CMV infection and was driven by the loss of naïve CD8 T cells; there was no increase in the absolute numbers of memory CD4 or CD8 T cells with aging in the absence of CMV. However, increases in the absolute size of the CD8^+^ and CD4^+^ effector T cell memory pools were observed with CMV infection. These CMV-reactive T cells are very “trigger-happy”, meaning that they produce pro-inflammatory cytokines in response to cross-reactive self or microbial antigens and may contribute to the chronic inflammation seen in many individuals as they age. Dr Nikolich-Zugich also presented results showing that many of the T cell precursors in unimmunized old mice survive by becoming “virtual memory” cells, with increased immediate cytokine secretion (another potential contributor to increased inflammation) but with reduced proliferative potential.

**Bonnie Blomberg(University of Miami)** described the effects of aging and chronic inflammation on B cell and antibody function. B cell numbers decrease with age, as does the amount and quality of antibodies produced in response to infections or vaccines. Dr. Blomberg studied the humoral immune response to influenza vaccine in elderly and young adults. She found that the anti-influenza serum antibody response to vaccine decreases with age and that this correlates with the percentage of switched memory B cells before the vaccine is given. An additional predictive biomarker for the antibody response was the activity of activation-induced cytidine deaminase (AID) in mitogen activated B cells *in vitro*. Unstimulated B cells from aged humans and mice also made more TNF than those from younger individuals. This TNF production was negatively correlated with B cell function and *in vitro* AID activity. The E2A-encoded transcription factor, E47, which is critical for B lymphopoiesis and AID function, class switch recombination, and affinity maturation of antibody, was also decreased in aged B cells. Overall, these observations suggested that changes in E47, AID, and TNF expression could be used as biomarkers to predict or follow functional B cell activity in various immune conditions, including aging.

In summary, the presentations and discussions in this session describe the changes in adaptive immunity observed during the aging process. Knowledge gaps and research opportunities were identified that could enhance our understanding of the role of aging in immune dysfunction and to mitigate or prevent age-related immune dysfunction.

### Session IV: Research Opportunities

- Document key changes in adaptive immune cells, such as terminally differentiated “trigger happy” T cells, leading to the production of inflammatory cytokines and loss of specificity with age.
- Determine whether the loss of immunologic space due to increases in memory and terminally differentiated T cells contributes to immunosenescence.
- Focus on defining biomarkers of immune senescence:
  - p16, SASP-associated cytokines, loss of CD28 expression, reduced telomerase activity, AID response to CpG, granzyme B in plasma, BAL, CSF, etc.
- Define the drivers of immunosenescence including:
  - Chronic or persistent infections – such as CMV or HIV,
  - Gut mucosa/microbiome changes with age,
  - Virus entry through the gut linked to age-related neurodegenerative diseases
  - Interactions of T cells with adipose tissue (and potentially other tissues).
- Develop therapeutic approaches to prevent or reverse immunosenescence including:
  - Preventative vaccines to prevent CMV and HIV infection,
  - Telomerase or CD28 gene therapy,
  - Blocking soluble cytokines such as TNF-alpha,
  - Modulating the microbiome of the gut and/or improving gap junctions,
  - Develop small molecule therapies or psychological or exercise interventions to reverse age-related changes and improve immune responses.
- Better understand the contribution of innate immune cells to inflammatory processes with aging and how to modulate for optimal responses to acute vs. chronic stressors.
- Define methods to modulate inflammatory effects in the tissue locally rather than to systemically modulate these effects

Overall, knowledge gaps and research opportunities were identified that could enhance our understanding of the role of aging in inflammatory processes contributing to various aging disorders.

Effective measures to mitigate or prevent age-related immune dysfunction need to be developed and could include vaccines to prevent chronic infections, blocking of soluble inflammatory cytokines, or modulating the microbiome of the gut. A better understanding of the mechanisms of age-related changes in the immune system leading to chronic inflammation and chronic disease are needed.

